# Prognostic and clinicopathological significance of TRIM21 in various cancers: A meta and bioinformatic analysis

**DOI:** 10.1097/MD.0000000000034012

**Published:** 2023-06-09

**Authors:** Feng Hu, Yan Liu, Feiyang Wang, Xinyi Fu, Xiangjun Liu, Zhenhong Zou, Bin Zhou

**Affiliations:** a Department of General Surgery, The Second Affiliated Hospital of Nanchang University, Nanchang, Jiangxi, People’s Republic of China; b The First School of Clinical Medicine of Nanchang University, Jiangxi Medical College of Nanchang University, Nanchang, Jiangxi, People’s Republic of China; c The Second School of Clinical Medicine of Nanchang University, Jiangxi Medical College of Nanchang University, Nanchang, Jiangxi, People’s Republic of China.

**Keywords:** bioinformatic analysis, meta-analysis, neoplasms, prognosis, TRIM21

## Abstract

Tripartite motif-containing protein 21 (TRIM21), a member of the ubiquitin ligase family, makes a significant contribution to the ubiquitination of multiple tumor marker proteins associated with tumor cell proliferation, metastasis and selective apoptosis. As the research further develops, an increasing number of studies have manifested that the TRIM21 expression level can be considered an indicator of cancer prognosis. However, the interrelationship between TRIM21 and multiple forms of carcinogens has not been demonstrated in a meta-analysis.

**Methods:**

We performed a systematic literature retrieval in various electronic databases including PubMed, Embase, Web of Science, Wanfang and China National Knowledge Infrastructure. Besides, the hazard ratio (HR) and the pooled relative risk (RR) were integrated in the assessment of cancer incidence and cancer mortality by Stata SE15.1. Additionally, we used an online database based on The Cancer Genome Atlas (TCGA) to further validate our results.

**Results:**

A total of 17 studies were included, totaling 7239 participants. High expression of TRIM21 was significantly correlated with better OS (HR = 0.74; 95% CI: 0.57–0.91; *P* < .001) and progression-free survival (PFS) (HR = 0.66; 95% CI: 0.42–0.91; *P* < .001). We found that high TRIM21 expression predicted significant impact on clinical characteristics like decreased lymph node metastasis (RR = 1.12; 95% CI: 0.97–1.30; *P* < .001), tumor stage (RR = 1.06; 95% CI: 0.82–1.37; *P* < .001) and tumor grade (RR = 1.07; 95% CI: 0.56–2.05; *P* < .001). However, TRIM21 expression had no significant impact on other clinical characteristics such as age (RR = 1.06; 95% CI: 0.91–1.25; *P* = .068), sex (RR = 1.04; 95% CI: 0.95–1.12; *P* = .953), or tumor size (RR = 1.14; 95% CI: 0.97–1.33; *P* = .05). Based on the Gene Expression Profiling Interactive Analysis (GEPIA) online analysis tool, TRIM21 was significantly downregulated in 5 cancers while significantly upregulated in 2 cancers, and the descending expression of TRIM21 predicted shorter OS in 5 cancers, worse PFS in 2 malignancies, while the elevated expression of TRIM21 predicted shorter OS and worse PFS in 2 carcinomas.

**Conclusions:**

TRIM21 could serve as a new biomarker for patients with solid malignancies and could be a potential therapeutic target for patients.

## 1. Introduction

Cancer refers to one of the leading causes of death globally that has conquered researchers’ attention for decades. According to the latest statistics, worldwide, an estimated 19.3 million new cancer cases and almost 10.0 million cancer deaths occurred in 2020.^[1]^ With the advance of research process, many tumor markers have been proven to play an important role in various cancers, but only a few have been used in clinical practice. Therefore, identifying valuable tumor biomarkers is essential for both cancer screening and the prognosis.

Tripartite motif containing protein 21 (TRIM21), also named as Ro52,^[2]^ is a member of the ubiquitin ligase group belonging to RING family which can be featured by the existence of a tripartite motif.^[3]^ Multiple studies have shown that the expression level of TRIM21 is reduced in the poor prognosis and the deterioration of malignancies, such as diffuse large B-cell lymphoma,^[2]^ renal cell cancer,^[4]^ hepatic cell cancer,^[5]^ breast cancer,^[6,7]^ and ovarian cancer.^[8]^ By contrast, in glioma^[9]^ and pancreatic cancer,^[10]^ patients with poor prognosis, the TRIM21 level is elevated, which indicates the additional function of TRIM21 in diverse types of cancers. Though present articles have manifested the relationship between TRIM21 and several malignancies, the significance of TRIM21 in prognosis has not been evaluated in meta-analysis. Therefore, we systematically collected and analyzed data from published literature to reveal the relationship between TRIM21 expression and overall survival (OS), progression-free survival (PFS), and clinicopathological features in cancer patients to assess the prognostic value of the possibly new biomarker TRIM21 in diverse types of cancers.

## 2. Materials and methods

### 2.1. Literature retrieval strategy

Web of Science, PubMed, Embase, China National Knowledge Infrastructure, and Wanfang databases are included in the scoepe of systematic computer literature retrieval. The supporting information are shown in the table, using the keywords: (“TRIM21” OR “Tripartite motif containing protein 21” OR “ubiquitin ligase”) AND (“cancer” OR “tumor” OR “neoplasm” OR “carcinoma” OR “malignancy”) AND “prognosis.” Besides, we also performed a manual screen to obtain potentially relevant studies.

### 2.2. Selection criteria

Eligible materials were selected into this meta-analysis based on the following criteria: the correlation between TRIM21 expression and OS and disease-free survival (DFS) in cancer patients; provided clinicopathological parameters; studies divided patients into 2 or more groups based on TRIM21 expression level; reported hazard ratio (HR) with 95% confidence interval (CI) or given sufficient data to estimate the HR with 95% CI. The exclusion criteria were: studies with insufficient data; letters, reviews, case reports, overlapping data, or expert opinions.

### 2.3. Date extraction and quality assessment

The extracted data and information were as follows: the first author name, year of publication, country, cancer type, number of patients, gender, method of assessing TRIM21 expression, cutoff value, follow-up times (months), outcome measures, analysis type, clinicopathological characteristics, HRs with 95% CI, prognostic value (OS, PFS), and *P* values. If the studies only provide the survival curve in figures, the estimated survival data were extracted by Engauge Digitizer.

### 2.4. Validation of bioinformatics database

Gene Expression Profiling Interactive Analysis (GEPIA), a web-based tool to deliver fast and customizable functionalities based on TCGA and GTEx data, was used. Survival plots of the correlation between TRIM21 expression and OS or DFS were retrieved as Kaplan–Meier curves based on different cancer datasets from the GEPIA online database. Median was set for cutoff value. Differential expression analysis between cancer and normal tissues was conducted based on GEPIA online analysis. All *P* value < .05 was regarded as statistically significant.

### 2.5. Statistical methods

All statistical analyses were carried out by Stata SE12.0. HRs with 95% CI for OS and DFS and relative risks (RRs) for clinicopathological parameters are included in the calculation. Heterogeneity was assessed among studies using χ^2^ tests and *I*^2^ statistics. We applied a random effects model to pool studies because of significant heterogeneity, as determined by the inconsistency index (*I*^2^ ≥ 50%) and χ^2^-test (*P* ≤ .10). The publication bias was evaluated by Begg funnel plot and Egger test, in which case *P* < .05 was considered significant.

## 3. Results

### 3.1. Main information of the enrolled studies

**Figure 1.**
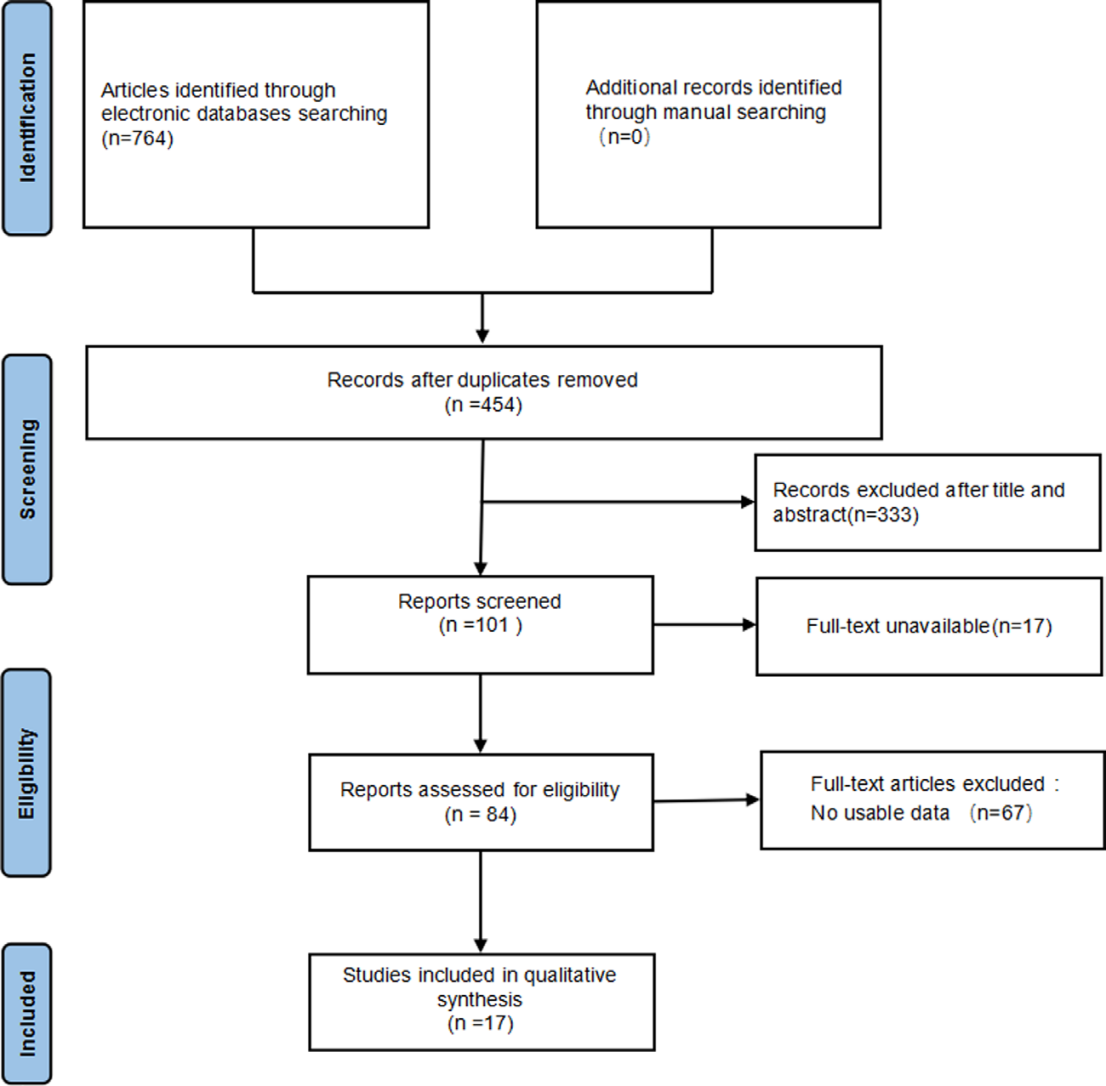
Flow chart of the study selection process.

In total, 764 studies were retrieved after searching keywords on PubMed, Web of Science, Embase, China National Knowledge Infrastructure, and Wanfang databases. Among these, 310 studies were removed as duplicates. A further 333 were excluded after screening the title and abstract, being studies not regarding cancers, studies performed on animals, review papers, or studies not evaluating the TRIM21 gene. A total of 84 studies were successively excluded for being unable to view full text and no available data were reported. Finally, 17 studies^[2,4–9,11–20]^ were included with total 7239 patients in this meta-analysis (Fig. 1). The flow chart maps out the number of studies identified, screened, included, and excluded.

The malignant neoplasms assessed in these studies included breast cancer, ovarian cancer, HCC (hepatocellular carcinoma), gastric cancer, pancreatic cancer, renal cell carcinoma, NSCLS (non-small cell lung cancer), HNSCC (head and neck squamous cell carcinoma), diffuse large B-cell lymphoma, and glioma. Sixteen studies were from China and 1 from Sweden. Ten were used to evaluate the HR of OS for cancer patients and 6 were used to evaluate the HR of both OS and DFS. The expression levels of TRIM21 gene were evaluated by immunohistochemistry in 10 studies, while several other studies adopted western blotting and Quantitative real-time PCR (qRT-PCR). The main information of the enrolled studies can be found in Table 1.

**Table 1:** Main characteristics of studies included in the meta-analysis.

Study	Country	Cancer type	Sample size	Follow-up	Tumor stage	Outcome measures	Analysis type	Gender (f/m)	NOS
Zhang ZH (2022)	China	HCC	198	167	NA	OS	U	NA	7
Zhang J (2021)	China	NSCLC	132	NA	100/32 (1/2–3)	NA	U	31/35	7
Yan G (2021)	China	RCC	157	150	NA	OS	U	NA	6
Xue CF (2017)	China	Ovarain cancer	1656	240	NA	OS	U	1656/0	6
Wang JL (2021)	China	GC	80	36	33/28/19 (2/3/4)	OS	U	23/57	7
Wang JL (2022)	China	Pancreatic cancer	87	36	27/26/27/7	OS	U	32/55	7
He D (2016)	China	Ovarain cancer	90	NA	42/48	OS	U	90/0	7
Zhou W (2018)	China	Breast cancer	169	74 1/2	67/87/15	OS/DFS	U/M	169/0	8
Zhao Z (2020)	China	Glioma	246	NA	NA	OS/DFS	U/M	NA	8
Sun J (2022)	China	Ovarain cancer	558	200	410/84/70/24	OS/DFS	U	558/0	6
Si W (2020)	China	Brest cancer	NA	300	NA	OS	U	NA	5
Jin Y (2019)	China	Brest cancer	2136	NA	1085/1051	NA	NA	2136/0	6
Ding Q (2015)	China	HCC	242	80	20/152/24/29	OS/DFS	U	31/211	8
Dai W (2015)	China	HCC	343	133	NA	OS	U	NA	6
Chuang C (2021)	China	HNSCC	518	200	NA	OS	U	NA	7
Chen X (2021)	China	RCC	246	60	NA	OS/DFS	U	44/79	6
Brauner S (2015)	Sweden	Lymphoma	381	100	NA	OS/DFS	U	NA	7

DFS = disease-free survival, GC = gastric cancer, HCC = hepatocellular carcinoma, HNSCC = head and neck aquamous cell carcinoma, NSCLC = non-small cell lung cancer, OS = overall survival, RCC = renal cell carcinoma.

### 3.2. Correlation of TRIM21 expression level with OS

**Figure 2.**
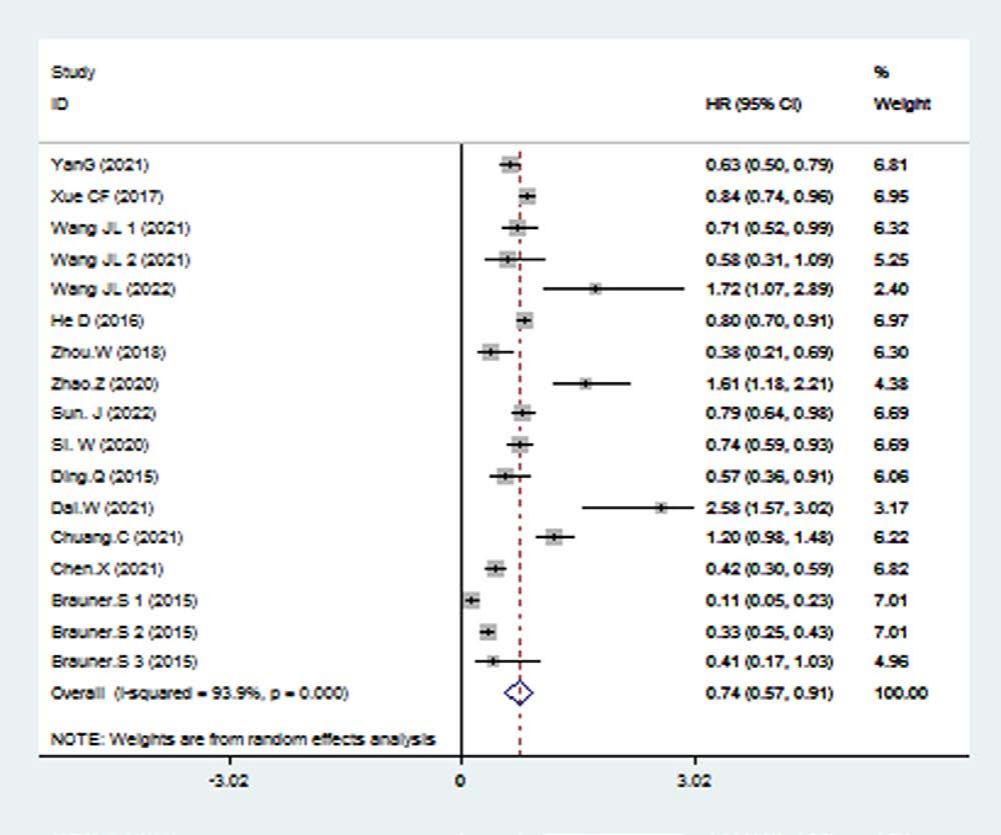
Forest plots to assess the OS analysis. OS = overall survival.

Data from the 17 studies were used for OS analysis with a random-effects model because of significant heterogeneity (*I*^2^ = 93.7%, *P* < .001). From the result, we could observe that compared with low TRIM21 expression, the high level of TRIM21 may predict better OS, with the pooled HR being 0.74 (95% CI: 0.57–0.91; *P* < .001; Fig. 2).

### 3.3. Subgroup analysis for OS

**Figure 3.**
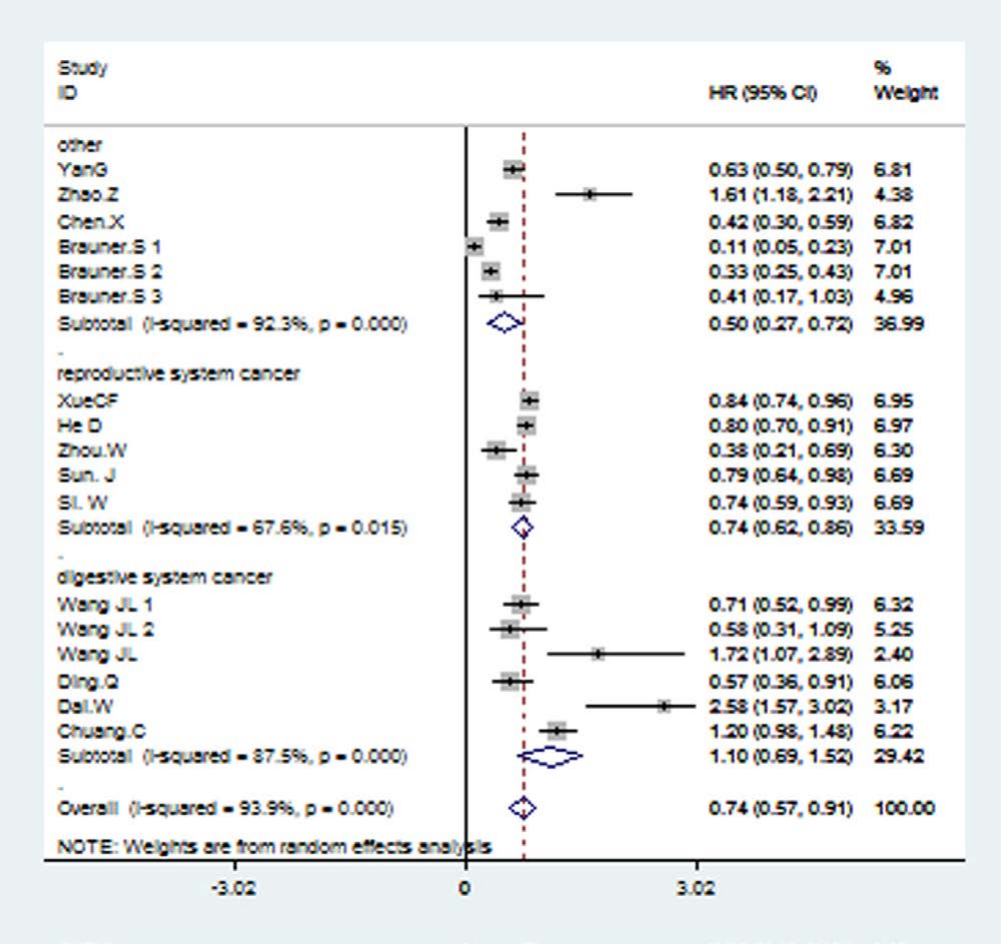
Forest plots for the subgroup analysis in different systems.

The included studies were analyzed by stratifying them according to subgroups of data source, pathology type, detection method, and tumor sites. Stratified analysis according to different tumor sites revealed a significant association between the high level of TRIM21 and better OS in reproductive system tumors (random effects model: combined HR = 0.74; 95% CI: 0.62–0.86; *P* = .015) and other systems (random effects model: combined HR = 0.50; 95% CI: 0.27–0.72; *P* < .001; Fig. 3). While a significant association between the high level of TRIM21 and worse OS was found in digestive system cancer (random effects model: combined HR = 1.10; 95% CI: 0.69–1.52; *P* < .001; Fig. 3); subgroup analysis according to pathological type showed a combined HR of 0.74 (95% CI: 0.63–0.85; *P* = .013) for adenocarcinoma and pooled HR (HR = 0.74; 95% CI: 0.49–1.00; *P* < .001) for another group (Supplementary Fig. 1, http://links.lww.com/MD/J126). In subgroup analysis data source (Supplementary Fig. 2, http://links.lww.com/MD/J127), a significantly better OS was observed both in reported data group (random-effects model: pooled HR = 0.79; 95% CI: 0.64–0.93; *P* < .001) and in data extracted from survival curve (random-effects model: pooled HR = 0.66; 95% CI: 0.43–0.89; *P* < .001). Finally, subgroup analysis according to different assay method showed that a high level of TRIM21 was associated with a significantly better OS in immunohistochemistry methods group (random-effects model: pooled HR = 0.50; 95% CI: 0.33–0.66; *P* < .001) and other group (random-effects model: pooled HR = 0.90; 95% CI: 0.061–1.20; *P* < .001) while worse OS in qRT-PCR group (random-effects model: pooled HR = 1.44; 95% CI: 0.90–1.99; *P* < .001) (Supplementary Fig. 3, http://links.lww.com/MD/J128).

### 3.4. Correlation of TRIM 21 expression level with PFS

**Figure 4.**
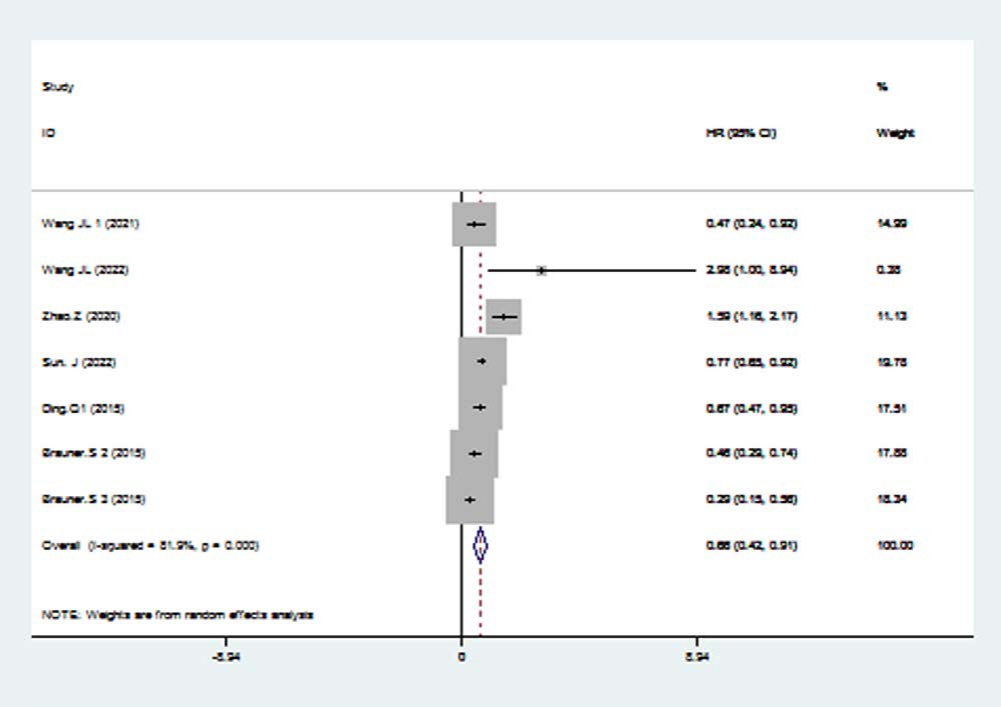
Forest plots of PFS analysis. PFS = progression-free survival.

HR for PFS was used in 6 studies, including 1594 patients The pooled HR confirmed a significant correlation between high TRIM21 gene and better PFS (HR = 0.66; 95% CI: 0.42–0.91; *P* < .001; Fig. 4). However, substantial heterogeneity was observed across studies, so the random-effects model was applied (*I*^2^ = 81.9; *P* < .001).

### 3.5. Association between the expression of the TRIM21 gene and clinical parameters

**Figure 5.**
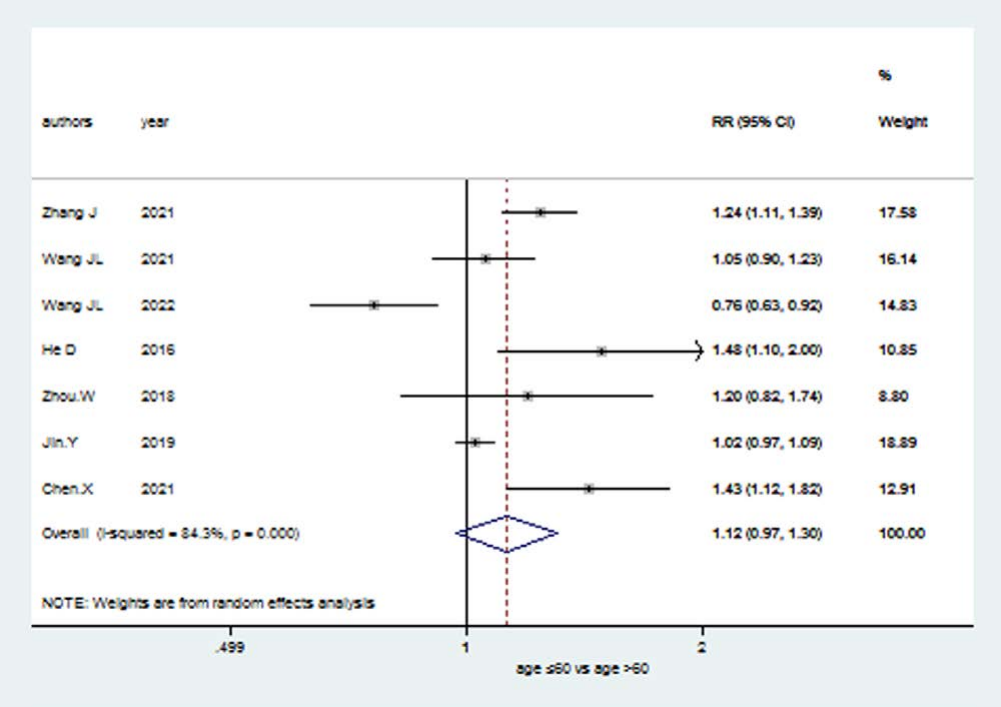
Forest plots for meta analysis between high TRIM21 gene and lymph node metastasis. TRIM21 = Tripartite motif-containing protein 21.

The relationship between the expression of TRIM21 gene and clinical outcomes were delineated in the meta-analysis. The information of Clinical Parameters was shown with pooled RRs and 95% CI (Table 2). We found that high TRIM21 expression predicted significant impact on clinical characteristics like lymph node metastasis (RR = 1.12; 95% CI: 0.97–1.30; *P* < .001; Fig. 5), tumor stage (RR = 1.06; 95% CI: 0.82–1.37; *P* < .001, random effects model; Supplementary Fig. 4, http://links.lww.com/MD/J129) and grade (RR = 1.07; 95% CI: 0.56–2.05; *P* < .001, random effects model; Supplementary Fig. 5, http://links.lww.com/MD/J130). However, no significant correlation was observed between high TRIM21 level and age (RR = 1.06; 95% CI: 0.91–1.25; *P* = .068, random effects model; Supplementary Fig. 6, http://links.lww.com/MD/J131), sex (RR = 1.04; 95% CI: 0.95–1.12; *P* = .953; Supplementary Fig. 7, http://links.lww.com/MD/J132), or tumor size (RR = 1.14; 95% CI: 0.97–1.33; *P* = .05, random effects model; Supplementary Fig. 8, http://links.lww.com/MD/J133).

**Table 2:** RRs and 95% CIs for patient survival or disease progression in association with TRIM21 expression in enrolled studies.

						Heterogeneity			
Parameters	Studies (n)	Number of patients (n)	RR	LCI	UCI	*P*	*I* ^2^	P_h_	Model
Age	6	3434	1.06	0.91	1.25	.068	51.40%	0.45	Random
Sex	5	787	1.04	0.95	1.11	.95	0.00%	0.41	Fixed
Lymph node metastasis	7	940	1.12	0.97	1.30	<.001	84.30%	0.13	Random
Tumor size	5	876	1.14	0.97	1.33	.05	57.90%	0.11	Random
Stage	7	3282	1.06	0.82	1.37	<.001	86.70%	0.65	Random
Tumor grade	7	3282	1.07	0.56	2.1	<.001	96.10%	0.83	Random

RR = relative risk, TRIM21 = Tripartite motif-containing protein 21.

### 3.6. Publication bias and sensitivity analysis

**Figure 6.**
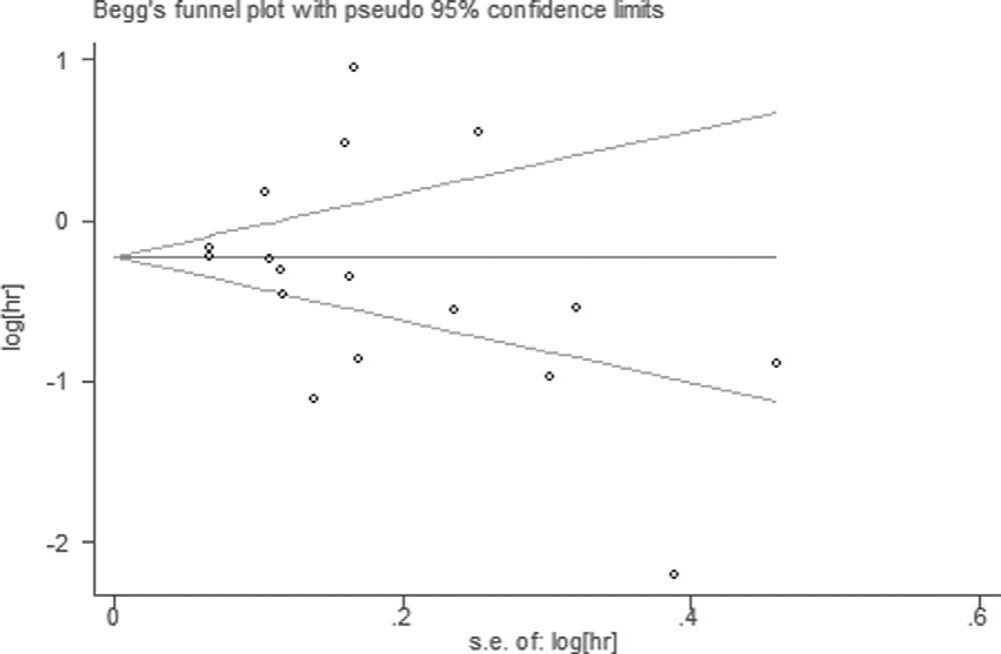
Begg funnel plots of the publication bias.

**Figure 7.**
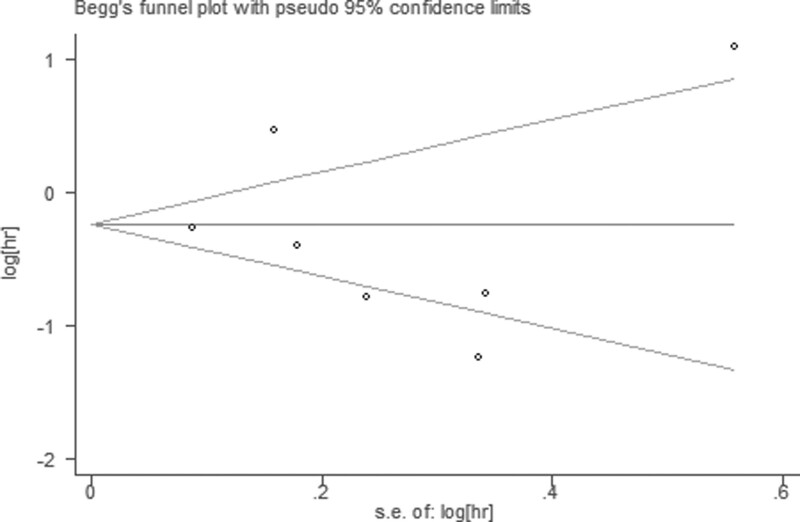
Begg funnel plots for Egger *P* value.

Publication bias was evaluated by Begg funnel plot and Egger test. Among 17 cohorts evaluating OS, no obvious asymmetry was observed in Begg funnel plots (Z = 1.77; *P*r >|Z| = 0.077) and the Egger *P* value (*P* = .396; Fig. 6), but asymmetry was found among 7 cohorts evaluating PFS (Z = 0.60; *P*r > |Z| = 0.548) and the Egger *P* value (*P* = .752; Fig. 7).

**Figure 8.**
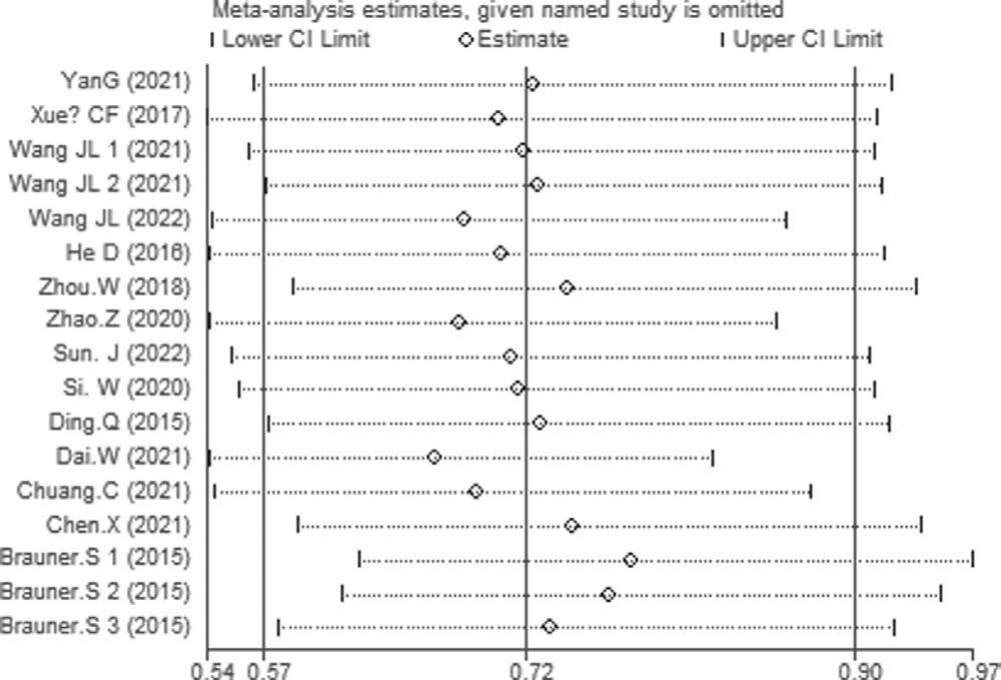
Sensitivity analysis of each included study.

A sensitivity analysis was applied to verify its accuracy of pooled HR of OS. The findings revealed that excluding any single research had no important effect on pooled HR, indicating the result relative (Fig. 8).

### 3.7. Validation of the results in the GEPIA database

**Figure 9.**
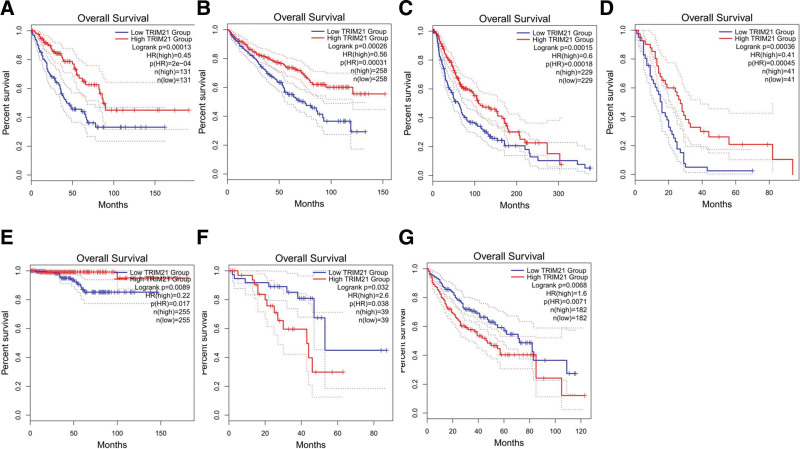
Validation of the prognostic effect of TRIM21 on cancer patient OS based on the GEPIA online database. OS = overall survival, TRIM21 = Tripartite motif-containing protein 21.

**Figure 10.**
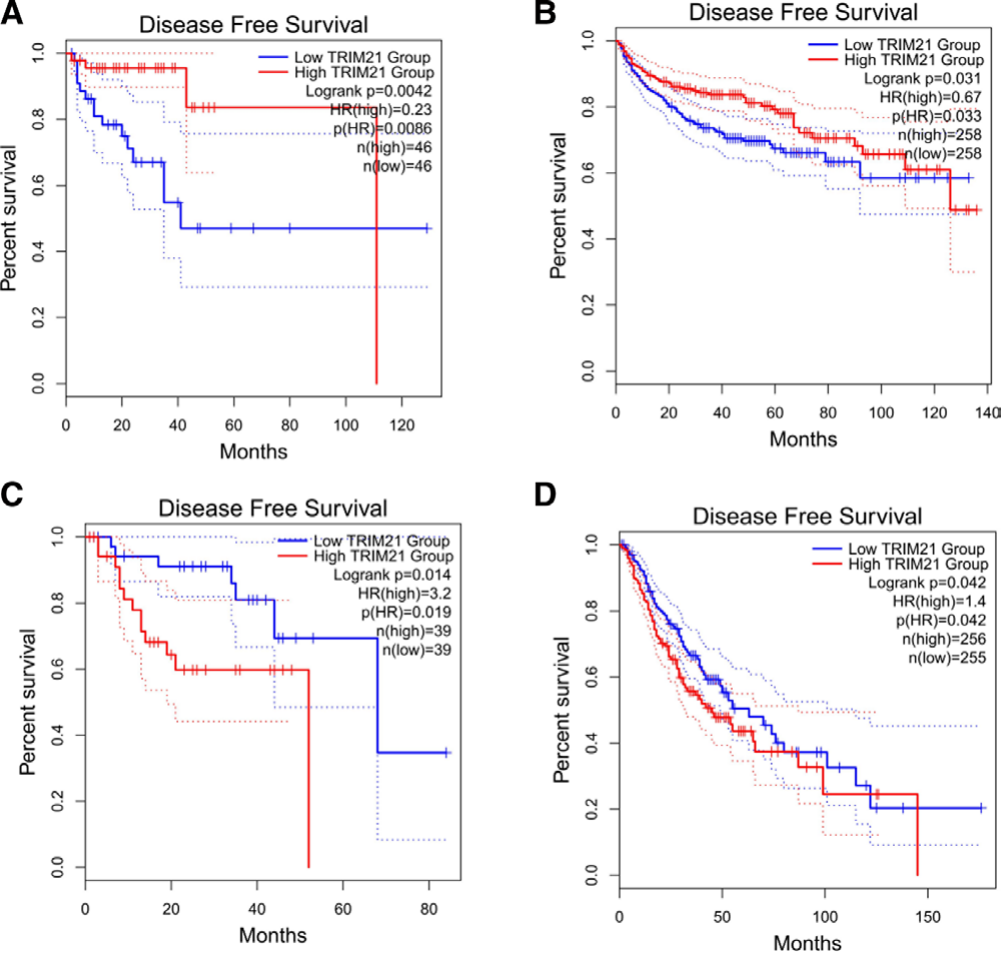
Validation of the prognostic effect of TRIM21 on cancer patient DFS based on the GEPIA online database. DFS = disease-free survival, GEPIA = gene expression profiling interactive analysis, TRIM21 = Tripartite motif-containing protein 21.

GEPIA online analysis tool was adopted to further strengthen our conclusion (http://gepia2.cancer-pku.cn/). In terms of TRIM21 dysregulation, low TRIM21 expression was identified in KIRC (Kidney renal clear cell carcinoma), MESO (Mesothelioma), SARC (Sarcoma), SKCM (Skin Cutaneous Melanoma), READ (Rectum adenocarcinoma) and THCA (Thyroid carcinoma). Regarding the association between and prognosis, decreased TRIM21 expression was correlated with worse OS in KIRC, MESO, SARC, SKCM, THCA (Fig. 9) and with worse PFS in KIRC and READ (Fig. 10). These results support our results and indicate that TRIM21 could be a novel prognostic biomarker for various cancers.

## 4. Discussion

TRIM21 gene is located on the short arm of chromosome 11 (11p15.5), the locus associated with malignancies in many organs. Previous studies have indicated that TRIM21 play important roles in inflammation,^[21]^ autoimmunity and cancer. TRIM21 was first discovered as an auto antigen associated with autoimmune diseases, such as SLE (Systemic Lupus Erythematosus).^[22]^ The up-regulation of TRIM21 can reduce cell proliferation and promote cell apoptosis in SLE, which indicated the TRIM21 could regulate the cell proliferation and apoptosis.^[23]^ After that, studies have shown pathogen infection such as viruses and bacteria can upregulate TRIM21 expression and further achieve its effective anti-pathogen protection by activating related transcription factor pathways, such as NF-κB, activator protein 1 (AP-1), interferon regulators IRF3, IRF5, and IRF7.^[24]^

Besides, the role of TRIM21 in various cancers has been observed in many studies, but the conclusions are inconsistent. These inconsistent outcomes might hint at dissimilar potential mechanisms that affect cancer recurrence. Like other E3 ligases, TRIM21 functions by ubiquitination of target substrates.^[25]^ A high expression of TRIM21 arrests the HCC cells cycle and inhibits cells proliferation by promoting ATG4B ubiquitylation,^[26]^ and suppresses the invasion of HCC cells by promoting β-catenin ubiquitylation and degradation.^[27]^ TRIM21 negatively regulates the NF-κB which is known as lymphoma proliferation and survival factor by the ubiquitylation and translocation of IKKβ to autophagosomes.^[28,29]^ Therefore, it might be the mechanism of TRIM21. High TRIM21 expression is associated with better prognosis in patients with diffuse large B-cell lymphoma (DLBCL).^[2]^ By contrast, high TRIM21 levels were associated with lower OS and poor prognosis in pancreatic cancer patients.^[10]^ The specific physiological role and detailed molecular mechanism by which TRIM21 contributes to tumor cells remain unclear. The exact functions of TRIM21 have not been fully understood and there are a series of mysteries that need to be explored and studied.

The results of the subgroup analysis showed that the relationship between TRIM21 expression levels and prognosis of patients was influenced by a variety of factors, including tumor types, the ethnicity of the study population, the method of detection and the source of the data analysis. Firstly, we performed subgroup analysis based on the different systemic origins of the tumors. The results showed that high levels of TRIM21 expression were significantly associated with better prognosis in patients with reproductive tumors or other cancers, but worse OS with digestive system cancers. In light of this, the prognostic value of TRIM21 may vary in different cancers because of different mechanisms of TRIM21. Secondly, we performed a subgroup analysis based on different pathological types to investigate the impact of TRIM21 expression levels on patient prognosis. The analysis showed that high levels of TRIM21 were significantly associated with better OS in adenocarcinoma, suggesting that detecting TRIM21 expression levels in patients with adenocarcinoma may help to predict their prognosis.

Our meta-analysis comprehensively and systematically reviewed the prognostic value of TRIM21 in various cancers. In this study, OS analysis revealed a pooled HR of 0.74, demonstrating that high TRIM21 expression is related with a better prognosis; however, the heterogeneity is significant (*I*^2^ = 93.7%; *P* < .001). PFS analysis also showed that increased TRIM21 expression is associated with better outcome significantly (HR = 0.76; *P* < .05). In addition, increased TRIM21 expression is negatively associated with lymph node metastasis with pooled RR (RR = 1.12; 95% CI: 0.97–1.30; *P* < .001), but no significant correlation was observed between TRIM21 and age, sex, tumor size, tumor grade or stage. GEPIA and TCGA databases were further used to validate our results as broadly as possible. Low TRIM21 expression levels were observed in KIRC, MESO, SARC, SKCM, READ, and THCA. What more, decreased TRIM21 expression was associated with worse OS in KIRC, MESO, SARC, SKCM, THCA, and with worse PFS in KIRC, READ. However, high TRIM21 expression levels were observed in LIHC (Liver hepatocellular carcinoma) and UVM (Uveal Melanoma), and elevated TRIM21 was associated with worse OS and PFS in these 2 cancers. Taken together, the research of TRIM21 may provide new ideas for the diagnosis and treatment of malignant tumors, and the research as a targeted treatment site of malignant tumors has achieved initial success. TRIM21 will bring new prospects for the clinical treatment of tumors.

There is no denying that several limitations existed in this meta-analysis. Firstly, the included tumor types had a narrow distribution range, mainly focusing on few cancer studies, which could not well represent the whole tumors. Secondly, the sample size was evenly distributed, and the difference in sample size would have some impact on HR. Thirdly, the TRIM21 expression level intercept was not uniform, and the difference between the low TRIM21 expression and the high TRIM21 expression would increase the heterogeneity of this study. Fourthly, the HR of some studies was extracted by KM survival curve, and there are errors in the extraction process, which will further affect the results of HR. Fifthly, this meta-analysis includes some retrospective studies. Therefore, these factors should be considered when drawing a conclusion. Finally, this study was nearly constrained to studies published in China, so our results may best illustrate the association between TRIM21 and Asian patients.

In conclusion, TRIM21 may affect the prognosis of cancer in some extent. It might be an effective indicator for predicting prognosis and tumor progression in the future. More clinical studies should be carried out to get a more accurate evaluation of the prognostic role of TRIM21 in patients with cancers.

## Author contributions

**Conceptualization:** Feng Hu, Yan Liu, Feiyang Wang, Xinyi Fu, Zhenhong Zou.

**Data curation:** Feng Hu, Yan Liu, Feiyang Wang, Xinyi Fu, Xiangjun Liu, Zhenhong Zou, Bin Zhou.

**Formal analysis:** Feng Hu, Yan Liu, Bin Zhou.

**Writing – original draft:** Feng Hu, Yan Liu, Feiyang Wang, Xinyi Fu, Xiangjun Liu.

**Writing – review & editing:** Feng Hu, Yan Liu, Zhenhong Zou, Bin Zhou.

## Supplementary Material
















