# Supplementary figures and images for: Prognostic and clinicopathological significance of TRIM21 in various cancers: A meta and bioinformatic analysis
Source: Medicine (Baltimore). 2023 Jun 9;102(23):e34012. doi: 10.1097/MD.0000000000034012 (PMC10256428; doi:10.1097/MD.0000000000034012)

Supplementary Figure 1 Forest plots for the subgroup analysis in different tumor types.

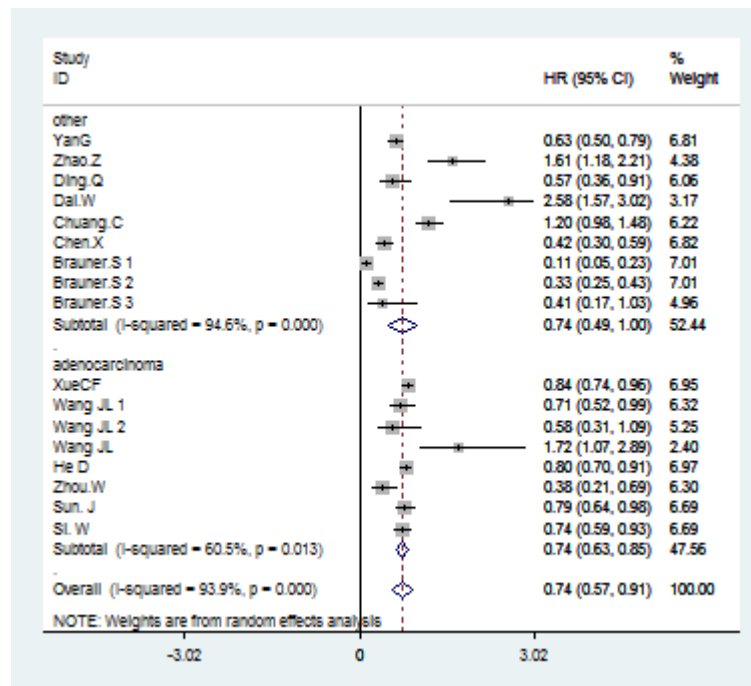

Supplement: Supplementary file 1 [file medi-102-e34012-s001.pdf]

Supplementary Figure 4 Forest plots for meta analysis between high TRIM21 gene and tumor stage.

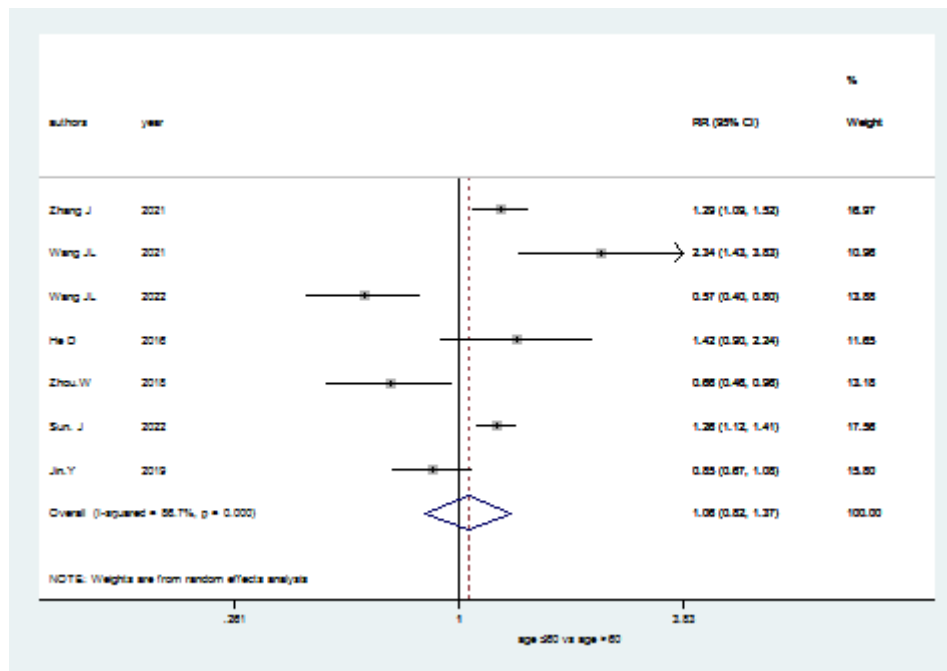

Supplement: Supplementary file 4 [file medi-102-e34012-s004.pdf]

Supplementary Figure 5 Forest plots for meta analysis between high TRIM21 gene and tumor grade.

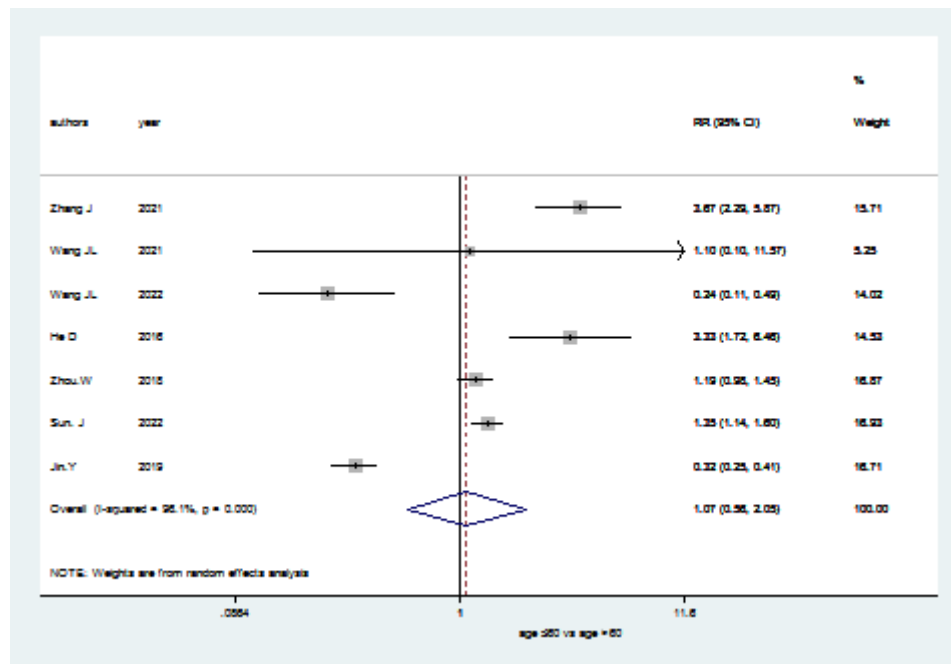

Supplement: Supplementary file 5 [file medi-102-e34012-s005.pdf]

Supplementary Figure 6 Forest plots for meta analysis between high TRIM21 gene and age.

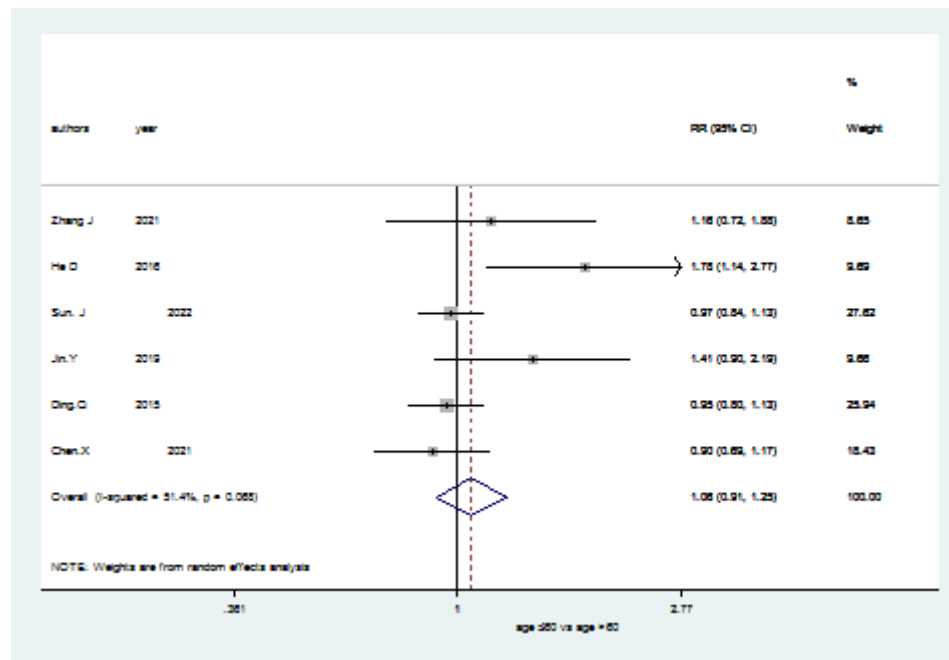

Supplement: Supplementary file 6 [file medi-102-e34012-s006.pdf]

Supplementary Figure 7 Forest plots for meta analysis between high TRIM21 gene and sex.

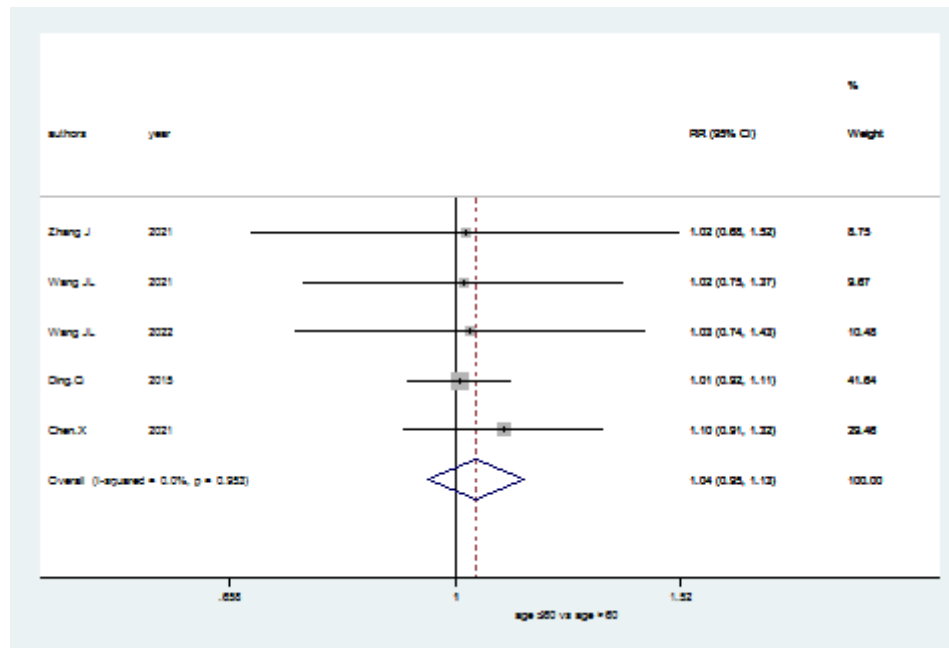

Supplement: Supplementary file 7 [file medi-102-e34012-s007.pdf]

Supplementary Figure 8 Forest plots for meta analysis between high TRIM21 gene and tumor size.

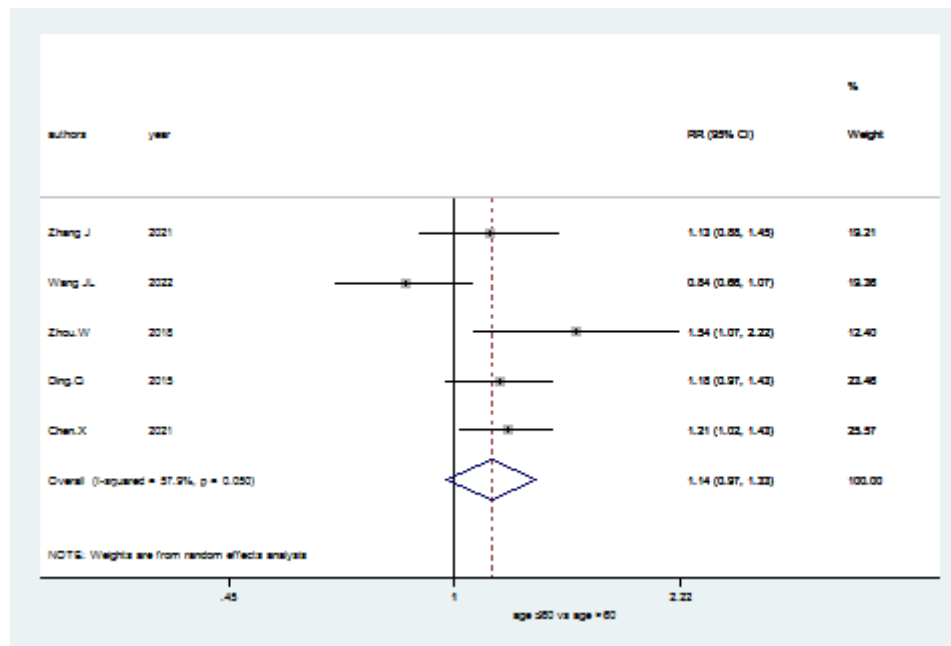

Supplement: Supplementary file 8 [file medi-102-e34012-s008.pdf]
