# Supplementary material for: Prognostic and clinicopathological significance of TRIM21 in various cancers: A meta and bioinformatic analysis
Source: Medicine (Baltimore). 2023 Jun 9;102(23):e34012. doi: 10.1097/MD.0000000000034012 (PMC10256428; doi:10.1097/MD.0000000000034012)

Supplementary Figure 2 Forest plots for the subgroup analysis in different data collection methods.

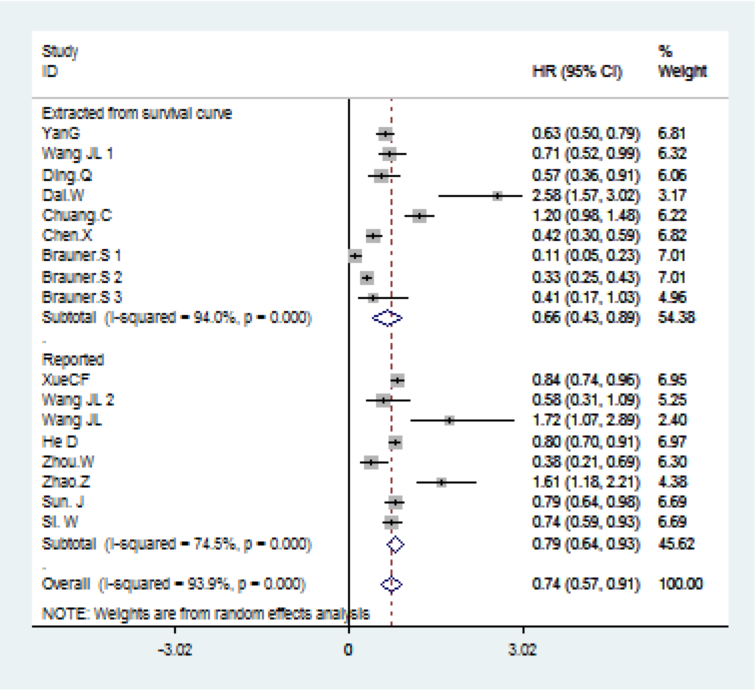

Supplement: Supplementary file 2 [file medi-102-e34012-s002.pdf]
